# TLK1B mediated phosphorylation of Rad9 regulates its nuclear/cytoplasmic localization and cell cycle checkpoint

**DOI:** 10.1186/s12867-016-0056-x

**Published:** 2016-02-09

**Authors:** Sanket Awate, Arrigo De Benedetti

**Affiliations:** Department of Biochemistry and Molecular Biology, Louisiana State University Health Sciences Center, 1501 Kings Highway, Shreveport, LA 71130 USA

**Keywords:** DNA damage response, Replication stress, TLK1, TLK1B, Rad9, pRad9 S328, WRN, γH2AX, 9-1-1 complex

## Abstract

**Background:**

The Tousled like kinase 1B (TLK1B) is critical for DNA repair and survival of cells. Upon DNA damage, Chk1 phosphorylates TLK1B at S457 leading to its transient inhibition. Once TLK1B regains its kinase activity it phosphorylates Rad9 at S328. In this work we investigated the significance of this mechanism by overexpressing mutant TLK1B in which the inhibitory phosphorylation site was eliminated.

**Results and discussion:**

These cells expressing TLK1B resistant to DNA damage showed constitutive phosphorylation of Rad9 S328 that occurred even in the presence of hydroxyurea (HU), and this resulted in a delayed checkpoint recovery. One possible explanation was that premature phosphorylation of Rad9 caused its dissociation from 9-1-1 at stalled replication forks, resulting in their collapse and prolonged activation of the S-phase checkpoint. We found that phosphorylation of Rad9 at S328 results in its dissociation from chromatin and redistribution to the cytoplasm. This results in double stranded breaks formation with concomitant activation of ATM and phosphorylation of H2AX. Furthermore, a Rad9 (S328D) phosphomimic mutant was exclusively localized to the cytoplasm and not the chromatin. Another Rad9 phosphomimic mutant (T355D), which is also a site phosphorylated by TLK1, localized normally. In cells expressing the mutant TLK1B treated with HU, Rad9 association with Hus1 and WRN was greatly reduced, suggesting again that its phosphorylation causes its premature release from stalled forks.

**Conclusions:**

We propose that normally, the inactivation of TLK1B following replication arrest and genotoxic stress functions to allow the retention of 9-1-1 at the sites of damage or stalled forks. Following reactivation of TLK1B, whose synthesis is concomitantly induced by genotoxins, Rad9 is hyperphosphorylated at S328, resulting in its dissociation and inactivation of the checkpoint that occurs once repair is complete.

**Electronic supplementary material:**

The online version of this article (doi:10.1186/s12867-016-0056-x) contains supplementary material, which is available to authorized users.

## Background

The Tousled (*Tsl*) gene was first identified in the plant *Arabidopsisthaliana.* Recessive *Tsl* mutants show defects in leaf and flower development [1]. This was proposed to be linked to a replicative defect during organogenesis, but it may also result from failure to protect the genome from DNA damage [2–4], resulting in developmental aberrations [5, 6]. Animal homologs of Tousled, known as Tousled like kinases (TLKs), are found from *Caenorhabditiselegans* to mammals. They are generally considered as genes of metazoans and are not found in yeast, although they are present in unicellular trypanosomes [7]. In mammals their activity is cell cycle regulated with maximal activity found in the S-phase. After many years of study, only a few direct “interacting” substrates of TLKs have been identified, namely the histone chaperone Asf1 [8], histone H3 [9], Rad9 [10], and Aurora B kinase [5]. As evident from their substrates, TLKs play a major role in chromatin assembly [10, 11], transcription [4, 12], DNA repair [3, 10, 13], and condensation of chromosomes at mitosis [5, 6]. In humans two structurally similar TLK genes (TLK1 and TLK2) with several splice variants have been identified. A splice variant of TLK1, TLK1B that lacks the first 237 amino acids was identified in our lab. TLK1 and TLK1B interact with similar substrates, are believed to have similar enzymatic functions and are often referred to as TLK1/1B. Our previous studies have shown that translation of TLK1B is induced by DNA damage through the activation of the mTOR-eIF4E pathway. We have shown that elevated expression of TLK1B promotes cell survival after irradiation (IR) or doxorubicin [13] and UV [3] by facilitating DNA repair and promoting chromatin assembly after repair. Expression of a dominant-negative mutant of TLK1B renders mammalian cells sensitive to IR [6]. Thus, the human homolog, TLK1B, has invoked interest because of its established role in cell survival after DNA damage [3, 9, 13]. Identification of Rad9 as a substrate for TLK1/1B attributes a direct role of TLK1/1B in DNA repair [14]. Our previous work suggests that TLK1/1B’s chaperone activity, independent of its kinase activity, helps in the recruitment of Rad9 at the break site. We had previously shown some evidence that TLK1/1B kinase activity is important for the dissociation of Rad9-Rad1-Hus1 (9-1-1) complex from a double stranded break (DSB) [14].

Rad9 plays a major role in DNA repair, cell cycle checkpoint and apoptosis. Aberrant Rad9 expression has been linked to breast, lung, thyroid, skin and prostate tumorigenesis [15]. Rad9 is a part of 9-1-1 heterotrimeric complex which is required for activation of ATR. Rad9, Rad1 or Hus1 KO mice are embryonic lethal [16, 17]. Loss of Rad9 produces a defect in ATR signaling and increases the sensitivity of the cells towards genotoxic stress [18]. In response to replication stress RPA directs the clamp loader RAD17–replication factor C (RFC) to load the 9-1-1 complex at the 5′ end of the double strand-single strand DNA junctions [19, 20]. Chromatin-bound 9-1-1 complex acts as a scaffold for the recruitment of various DNA repair proteins and polymerases at the DNA damage break site. It ensures filling of gaps and efficient repair of DNA [21, 22]. Recently it has been shown that 9-1-1 complex is required for the recruitment of WRN protein at stalled replication forks and this interaction is important for the fork recovery [23]. WRN belongs to the RecQ family of DNA helicases. Loss of WRN gives rise to a genetic disease known as Werner syndrome (WS). It is characterized by pre-mature ageing and pre-disposition to cancer [24, 25]. Cells derived from the WS patients show a prolonged S-phase, a reduced life-span, and an increase in genomic instability [26, 27]. It has been shown that WRN stabilizes the stalled replication forks and the loss of WRN leads to the fork collapse and increase in DSBs that are repaired through recombination [28]. WRN interacts with the 9-1-1 complex to maintain genomic stability by preventing accumulation of DSBs at the damaged forks [23].

Activation of the DNA damage induced checkpoint mediates rapid and transient inhibition of TLK activity. This transient inhibition in response to DNA damage requires ATM and Chk1 function. Chk1 directly phosphorylates TLK1 at S695 which is equivalent to S457 of TLK1B [29]. Once TLK1/1B regains its kinase activity it phosphorylates Rad9 at S328 [10] and T355 [30]. Rad9 S328 phosphorylation follows the pattern of TLK1/1B activity wherein it is inhibited immediately after DNA damage and gets phosphorylated when TLK1/1B regains its activity [14, 31]. The reason for this transient inactivation of TLK1/1B still remains a question as there is a lack of direct evidence for the role of this inhibitory phosphorylation with regards to its effect on Rad9 and ATR mediated cell signaling. In order to answer this question we have made an S457A mutant (Mut) of TLK1B that lacks the inhibitory phosphorylation site. In this study we show that Mut TLK1B remains active in the presence of DNA damage, and cells overexpressing it display an altered cell cycle checkpoint and delay in cell cycle progression upon recovery from hydroxyurea (HU). Mut TLK1B overexpressing cells show an increase in phosphorylation of Rad9 at S328. In response to HU mediated replication arrest, Mut TLK1B overexpressing cells show a massive reduction in the formation of Rad9 foci and reduced association of Rad9 with the chromatin. Mut TLK1B expressing cells show reduced association of Rad9 with Hus1 and WRN suggesting an early dissociation of 9-1-1 complex. In response to HU, these cells show an increased accumulation of DSBs which are marked by an increase in association of p-ATM (S1981) and γ-H2AX with the chromatin. Cellular fractionation data of Mut TLK1B overexpressing cells showed an increase in the accumulation of total Rad9 and p-Rad9 (S328) in the cytoplasm. Furthermore, studies with Rad9 S328D phosphomimetic mutant suggest that the phosphorylation of Rad9 at S328 alone is sufficient to prevent Rad9 localization into the nucleus. Our results indicate that in response to replication arrest, transient inhibition of TLK1/1B is crucial to maintain localization of Rad9 into the nucleus at damage sites.

## Results

### Mut TLK1B increases phosphorylation of Rad9 at S328 both in presence and absence of DNA damage

**Fig. 1:**
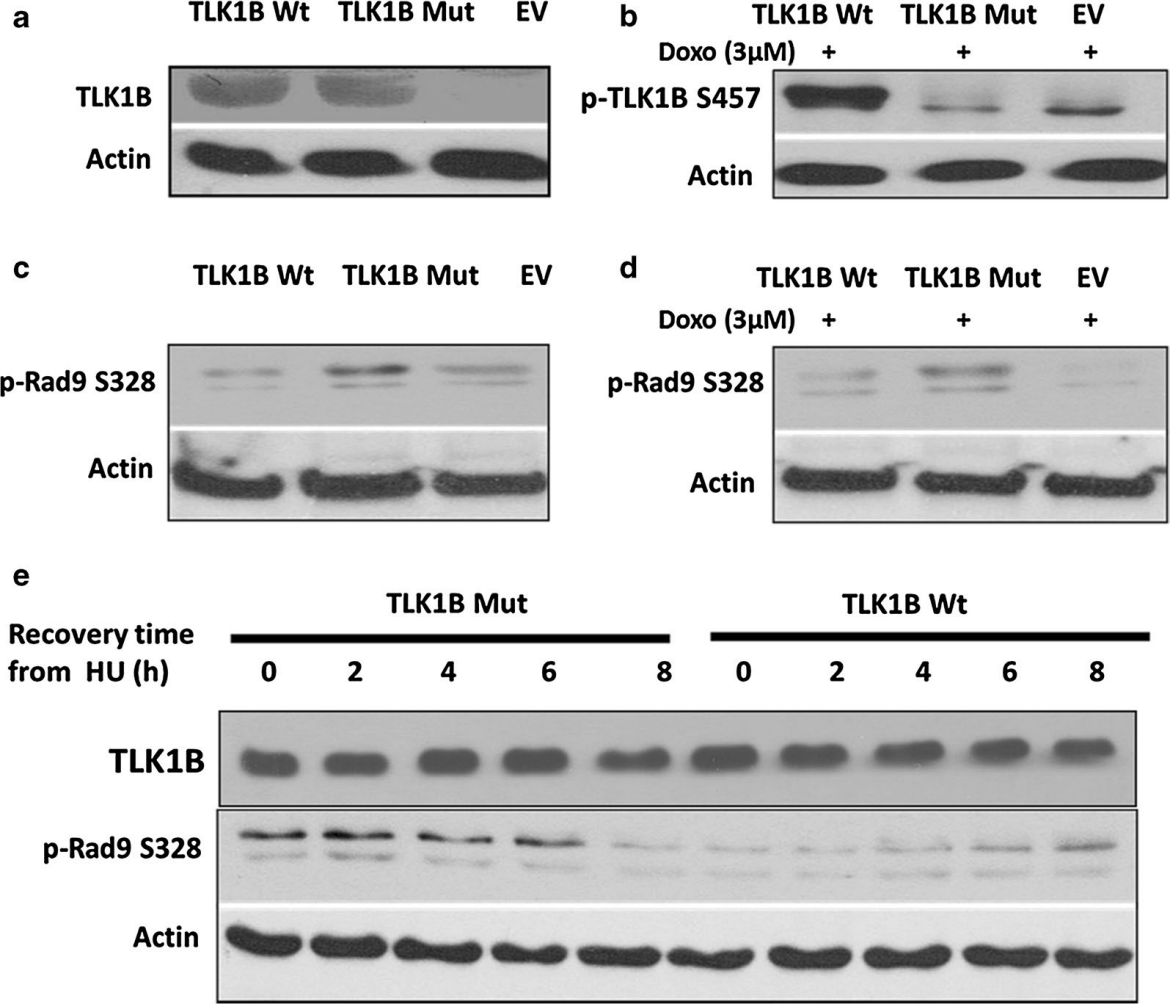
**a** Overexpression of Wt TLK1B and Mut TLK1B in stably transfected HEK293 cells. **b** TLK1B Wt and Mut cells or empty vector controls (EV) were treated for 2 h with doxo to promote the Chk1-dpendent phosphorylation of TLK1B (S457). TLK1B S457 phospho specific antibody recognizes only the overexpressed Wt TLK1B and not the Mut TLK1B. Note that the p-TLK1B *band* seen in the Mut lane corresponds to the endogenous TLK1B and not the transfected TLK1B Mut. **c** The basal phosphorylation of Rad9 (S328) is enhanced in cells expressing Mut TLK1B with respect to EV or cells expressing Wt TLK1B. **d** The phosphorylation of Rad9 (S328) persists in damage resistant Mut TLK1B expressing cells treated with doxo. **e** Pattern of Rad9 (S328) phosphorylation after recovery from HU in the cells expressing Mut TLK1B or Wt TLK1B. Note that TLK1B overexpression is unaffected by HU treatment in these cells

We generated a S457A TLK1B mutant which lacks the Chk1 inhibitory phosphorylation site and cloned it into the pIRES2-EGFP vector. We then generated HEK293 stable cell lines overexpressing mutant (Mut) and wild-type (Wt) TLK1B, after selection for high GFP positive cells (methods). Figure 1a confirms the overexpression of Wt and Mut TLK1B in HEK293 cells. Further, after treatment with doxorubicin (doxo, topoisomerase II inhibitor that causes accumulation of DSBs upon which Rad9 is loaded) Wt TLK1B shows an increase in phosphorylation at S457 whereas the phospho-specific antibody doesn’t recognize the overexpressed Mut TLK1B (Fig. 1b). The mutant and empty vector (EV) show a slight band of p-TLK1B S457 attributable to the endogenous TLK1B that is translationally induced after doxo treatment [9, 32]. In comparison with the Wt and EV, the basal level of Rad9 phosphorylation is elevated in the Mut TLK1B expressing cells (Fig. 1c). In the Mut TLK1B expressing cells the phosphorylation of Rad9 is not suppressed after DNA damage, either with doxo (Fig. 1d) or HU (Fig. 1e), which suggests that the Mut kinase is active even in the presence of DNA damage. In Wt cells, Rad9 phosphorylation at S328 is initially very low and progressively increases by 6–8 h of recovery from HU (Fig. 1e). In Mut TLK1B cells, the phosphorylation of Rad9 is elevated after release from HU but decreases after 8 h of recovery, possibly due to the activation of a phosphatase. HU depletes the deoxyribonucleotide pools that results in replication fork stalling, leading to recruitment of Rad9.

### Cells expressing Mut TLK1B show defects in formation of Rad9 foci

**Fig. 2:**
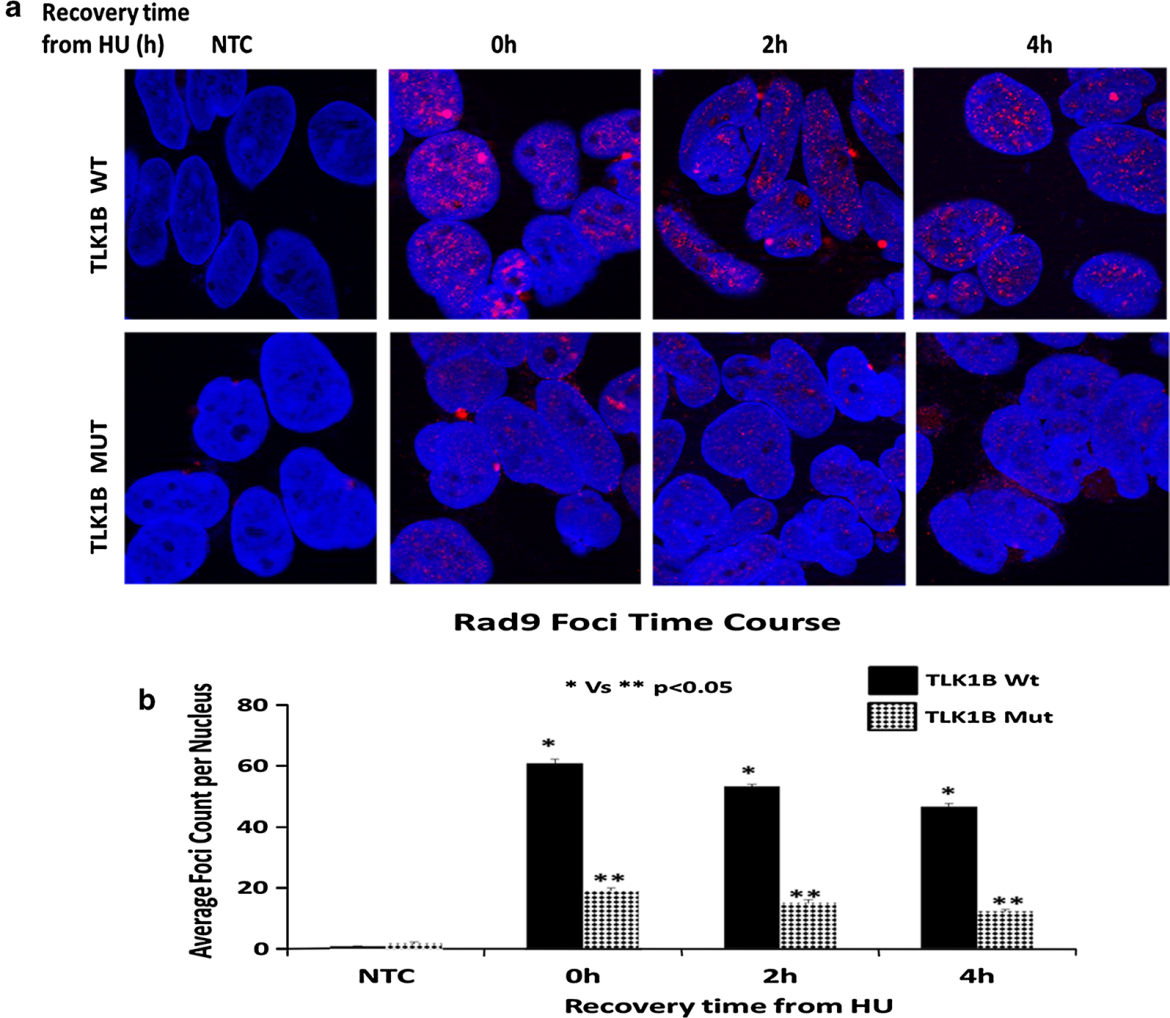
**a** Rad9 foci in the TLK1B Wt and Mut overexpressing cells were examined after removing the soluble proteins and fixing chromatin bound proteins (methods). **b** Quantitative analysis of the Rad9 foci per cell averaged over 100 cells per data point. All the experiments were performed independently at least three times. Values are mean ± SE

When cells are exposed to replication stress Rad9 protein is redistributed to form discrete nuclear foci at the sites of DNA damage [33]. The recruitment of the 9-1-1 complex at the sites of DNA damage can be visualized by looking at the formation of Rad9 foci [34]. We showed that in response to DNA damage Rad9 S328 phosphorylation persists in the cells overexpressing Mut TLK1B. We wanted to examine whether TLK1B mutant cells show any defects in formation of Rad9 foci. In order to look at the Rad9 foci associated with chromatin, soluble proteins were removed before fixation (as described in “Methods” section). In comparison with the Wt, cells expressing Mut TLK1B showed a drastic reduction in the formation of Rad9 foci. The Wt cells had much brighter and greater number of Rad9 foci (Fig. 2a, b) in comparison with the Mut TLK1B expressing cells. When the soluble proteins were not removed before fixation we saw an increased accumulation of Rad9 in the cytoplasm in the Mut TLK1B expressing cells (Additional file 1: Figure S1).

### Cells expressing Mut TLK1B show reduction in association of Rad9 with the chromatin

**Fig. 3:**
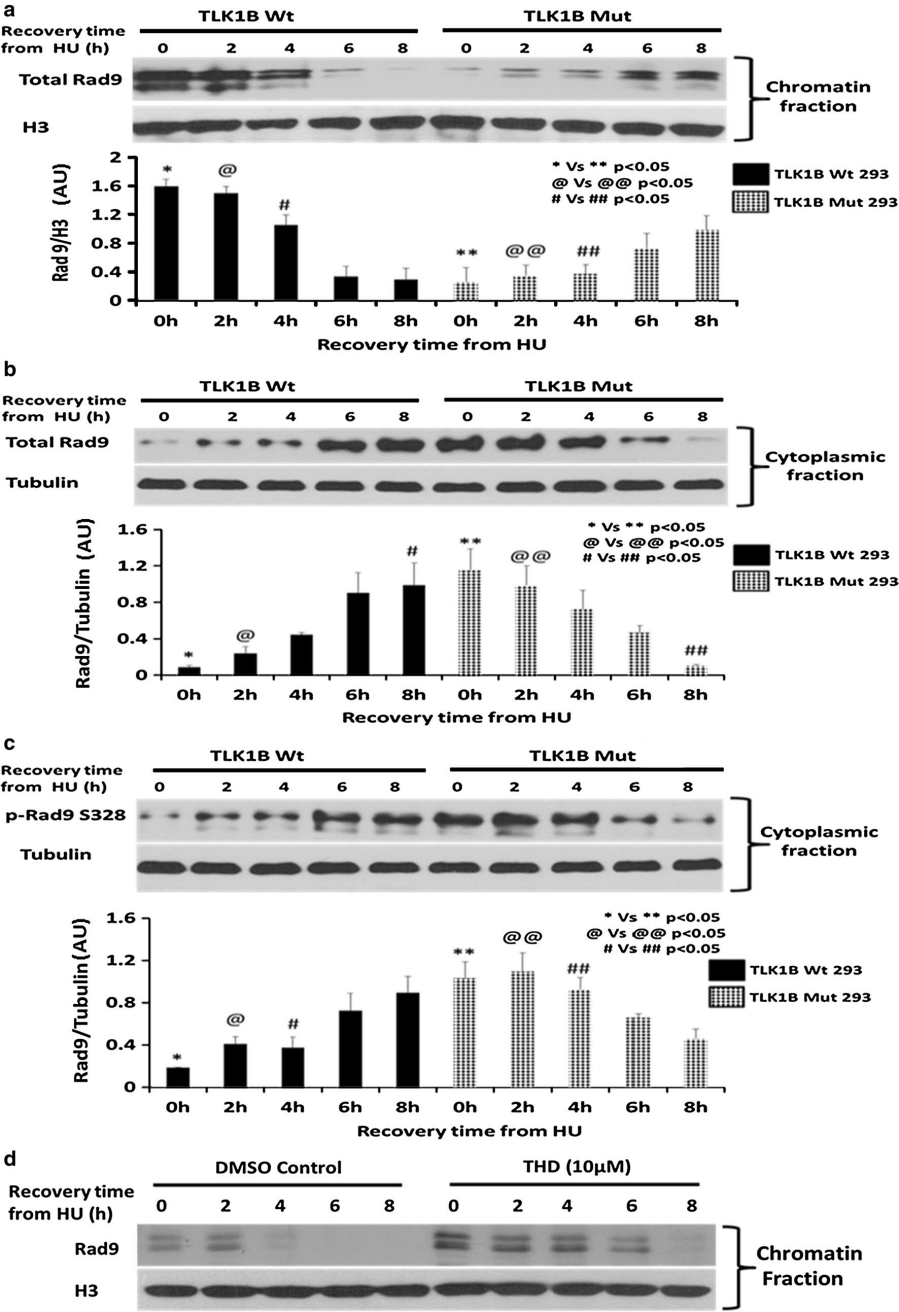
**a** Distribution of Rad9 in the chromatin-bound fraction in Wt and Mut TLK1B expressing cells in response to treatment and recovery from HU. Values are mean ± standard error for n = 3. **b** Distribution of Rad9 in the cytoplasmic fraction in Wt and Mut TLK1B expressing cells. Note that the Rad9 in the cytoplasm appears as a single band, but in the chromatin fraction appears as multiple bands. Rad9 gets multiply phosphorylated only once it gets loaded onto the chromatin. Values are mean ± SE for n = 3. **c** Distribution of p-Rad9 (S328) in the cytoplasmic fraction in Wt vs Mut TLK1B expressing cells. Note that p-Rad9 (S328) is predominantly cytoplasmic and is elevated in Mut TLK1B expressing cells during replication stress. Values are mean ± standard error for n = 3. **d** Distribution of Rad9 in the chromatin-bound fraction in HEK293 cells in response to treatment and recovery from HU in the presence or absence of TLK inhibitor (THD)

It has been shown that replication stress stimulates the association of Rad9 with the chromatin [35]. We next wanted to see if this phosphorylation may result in early dissociation of Rad9 from the chromatin in these cells. To study association and dissociation kinetics of Rad9 in the cells expressing Wt and Mut TLK1B we performed chromatin fractionation assay after treating cells with HU and allowing them to recover. In the Wt TLK1B expressing cells Rad9 was largely associated with the chromatin fraction at recovery time t = 0 (Fig. 3a). Rad9 was found in the chromatin bound fraction until 4 h of recovery period. However at around 6 to 8 h of recovery time the Rad9 levels decreased in the chromatin bound fraction. A very different pattern was seen in the Mut TLK1B expressing cells (Fig. 3a). These cells had very low levels of Rad9 bound to the chromatin fraction until 4 h of recovery period (significantly lower than Wt expressing cells). However after 6 h of recovery time the Rad9 levels increased in the chromatin bound fraction. We believe that this is due the accumulation of DNA damage (including DSBs) at this time, and that this may trigger a mechanism for the re-import of Rad9 into the nucleus that is independent on S328 phosphorylation by TLK1B.

### Cells expressing Mut TLK1B show an increased accumulation of Rad9 and p-Rad9 S328 in the cytoplasmic fraction

**Fig. 4:**
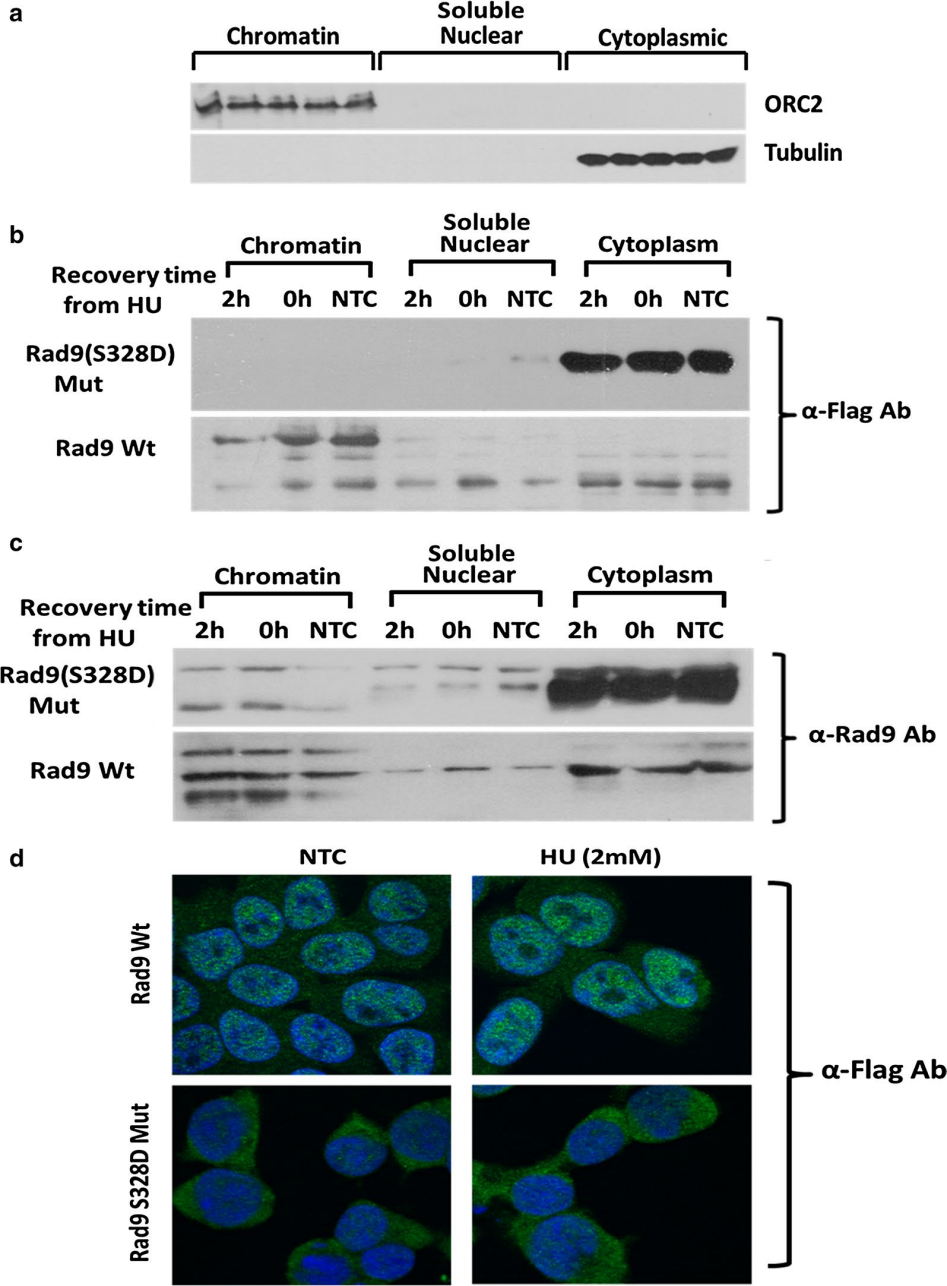
**a** Verification of fractionation procedure by probing for ORC2 which is chromatin-bound and tubulin which is cytoplasmic. **b** α-Flag Ab detects only the overexpressed flag tagged Wt and Rad9 (S328D) mutant. **c** α-Rad9 Ab detects both the endogenous and the overexpressed Wt and Rad9 (S328D) mutant. **d** Immunolocalization shows that the Rad9 (S328D) mutant localizes primarily in the cytoplasm in contrast to the WT protein which is mostly nuclear

Through fractionation we obtained cytoplasmic, soluble nuclear and chromatin bound proteins. Correct distribution was confirmed by immunoblots using tubulin and ORC2 antibodies (Fig. 4a). We expected that the phosphorylation of Rad9 at S328 would cause the release of Rad9 from chromatin fraction into the soluble fraction. When we analyzed the cytoplasmic fraction we saw a reciprocal distribution where the decrease of Rad9 from the chromatin fraction (Fig. 3a) corresponded with its increase in the cytoplasmic fraction (Fig. 3b) and vice versa. It should be noted that Rad9 in the cytoplasm appears as a single band, but gets phosphorylated on multiple residues once it gets loaded onto the chromatin, resulting in the appearance of multiple bands [36]. In the Wt TLK1B expressing cells until 2–4 h of recovery time, low levels of Rad9 were present in the cytoplasmic fraction as most of it was bound to the chromatin. However, around 6 h of recovery in these cells Rad9 levels decreased in the chromatin bound fraction and correspondingly increased in the cytoplasmic fraction. In the Mut TLK1B expressing cells, most of Rad9 was found in the cytoplasm until 4 h of recovery; however, around 6–8 h of recovery time Rad9 levels in the cytoplasm gradually decreased and correspondingly increased in the chromatin bound fraction. During replication stress Mut TLK1B expressing cells had increased levels of p-Rad9 S328 (Fig. 3c) in the cytoplasm. The pattern of Rad9 S328 phosphorylation in the cytoplasmic fraction exactly matched the pattern of total Rad9 (Fig. 3b) suggesting that the phosphorylation of Rad9 at S328 may be responsible for its accumulation in the cytoplasm. Previously using an adeno-HO-mediated cleavage system in MM3MG cells, we had shown that overexpression of kinase dead TLK delays the release of Rad9 and Rad17 upon repair of the DSB [14]. In our previous study we identified specific TLK inhibitors that prevent the TLK-mediated phosphorylation of Rad9 at S328 and cause defects in checkpoint recovery [31]. Mut TLK1B overexpressing cells, showing an increased TLK1B activity, showed an early dissociation of the Rad9 from the chromatin. We next wanted to examine if inhibition of TLKs with specific inhibitor Thioridazine hydrochloride (THD) leads to an increase in association of Rad9 with the chromatin. As expected, we saw an increased association of Rad9 onto the chromatin in the presence of THD (Fig. 3d). Also Rad9 remained bound onto the chromatin for longer time in the presence of the THD (Fig. 3d).

### Phosphomimetic Rad9 S328D mutant is sufficient to accumulate Rad9 in the cytoplasm

We next wanted to examine if phosphorylation of Rad9 at S328 is sufficient to alter the nuclear localization of Rad9. In order to do so we generated a phosphomimetic flag-tagged Rad9 S328D mutant (as described in “Methods” section). Fractionation was performed on the cells overexpressing the flag-tagged Rad9 S328D mutant and flag-tagged Wt Rad9. These cells were incubated with or without HU. After fractionating the cytoplasmic, soluble nuclear and chromatin bound proteins the distribution of Rad9 was examined by immunoblotting with either anti-flag antibody to specifically detect overexpressed flag-tagged Wt or Mut Rad9 (Fig. 4b) or anti-Rad9 antibody to detect both the endogenous and overexpressed Rad9 (Fig. 4c). Immunoblotting with anti-flag antibody showed that the overexpressed Rad9 S328D mutant was localized exclusively in the cytoplasm while the overexpressed Wt Rad9 was present in all the three fractions (Fig. 4b). These results confirm that the phosphorylation of Rad9 at S328 is sufficient to accumulate Rad9 in the cytoplasm. As previously mentioned multiple Rad9 bands appear in the chromatin fraction due to phosphorylation of Rad9 at multiple sites. Cellular fractionation results were confirmed by immunolocalization (as described in “Methods” section), which showed that the flag-tagged Rad9 (S328D) localized to the cytoplasm whereas the Wt Rad9 was nuclear (Fig. 4d).

### Phosphomimic Rad9-T355D does not affect its nuclear/cytoplasmic localization

Scott Davey`s group has recently found Rad9 T355 as another phosphorylation site of TLK1 [30]. Interestingly T355 lies next to nuclear localization sequence (NLS), which starts at the residue 356 and ends at 364. Bioinformatics analysis with cNLS mapper predicted that Rad9 is localized exclusively in the nucleus, and T355D mutation in Rad9 would cause a partial cytoplasmic accumulation. Our cellular fractionation data of Mut TLK1B overexpressing cells showed an increase in the accumulation of total p-Rad9 (T355) in the cytoplasm but it was unclear if this simply reflected the total Rad9 level, i.e., phosphorylated also at S328 (Additional file 2: Figure S2A). We next wanted to examine if phosphorylation of Rad9 at T355 can alter the nuclear localization of Rad9. In order to do so we generated a phosphomimetic flag-tagged T355D mutant. The Rad9 T355D mutant was also found in the chromatin and nucleoplasmic fractions, although the chromatin to cytoplasmic ratio for the T355D mutant was not identical to the wt Rad9 (Additional file 2: Figure S2B).

### Mut TLK1B expressing cells show reduced association of Rad9 with Hus1 and WRN

**Fig. 5:**
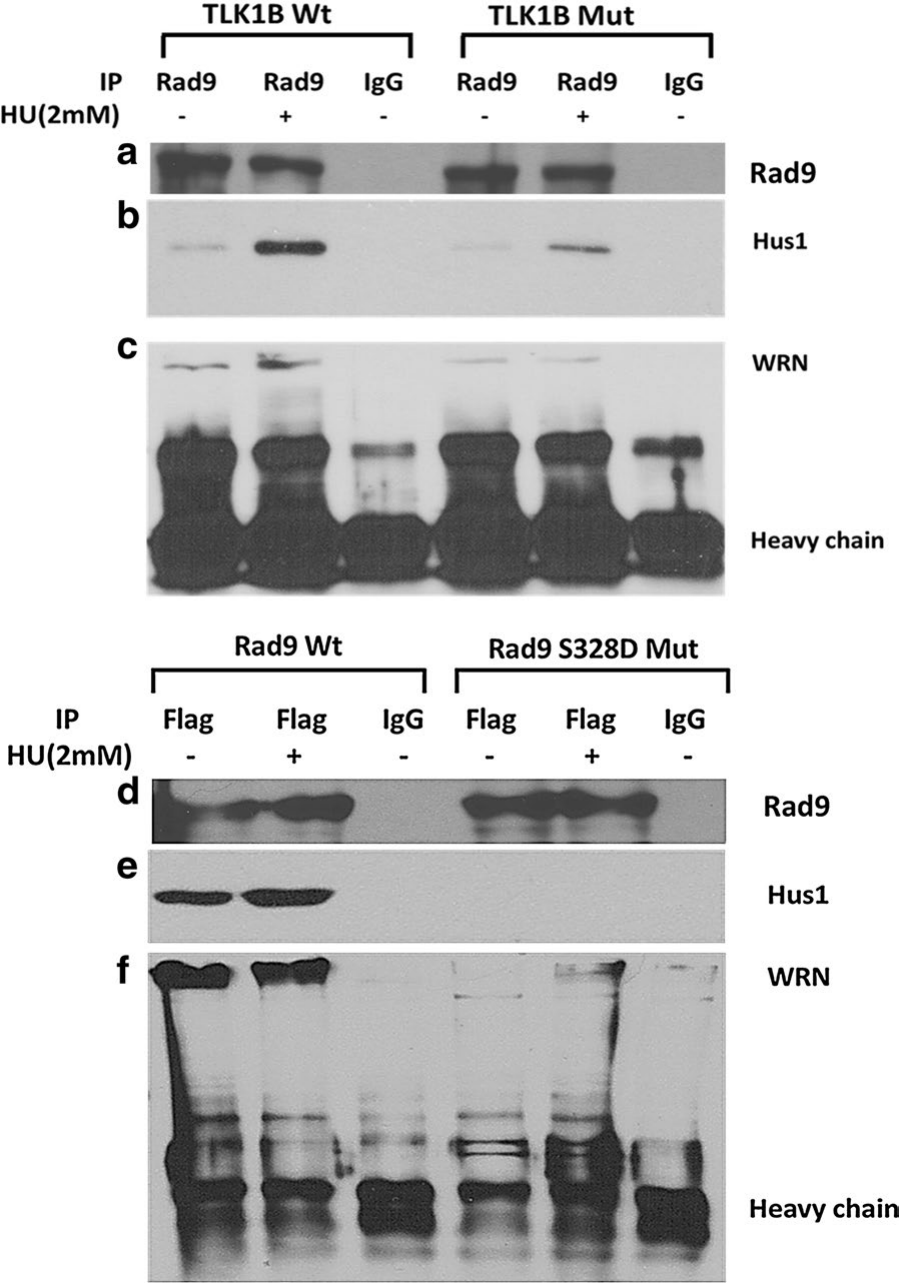
**a** Immunoprecipitation of Rad9 in Wt and Mut TLK1B expressing cells. **b** HU treatment results in increased formation of 9-1-1 complex as indicated by the increased association of Rad9 with Hus1. This interaction is greatly diminished in Mut TLK1B expressing cells. **c** Co-immunoprecipitation of WRN with Rad9 is also is greatly diminished in Mut TLK1B expressing cells. **d** Immunoprecipitation of overexpressed flag tagged Rad9 in Wt and (S328D) Mut Rad9 expressing cells. **e** Co-immunoprecipitation of Hus1 with overexpressed flag-Rad9 is diminished in Rad9 S328D Mut expressing cells. **f** Co-immunoprecipitation of WRN with overexpressed flag-Rad9 is diminished in Rad9 S328D Mut expressing cells

Stable 9-1-1 complex is required for the recruitment of WRN at the stalled replication forks. Replication stress stimulates the interaction of Rad9 with Hus1 [37] and WRN [23]. Inactivation resistant Mut TLK1B accumulates Rad9 in the cytoplasm which would affect recruitment of WRN at stalled replication forks. In order to examine the stability of the 9-1-1 complex and WRN recruitment we examined the interaction of Rad9 with Hus1 and WRN in the Wt and Mut TLK1B expressing cells. Cell lysates were immunoprecipitated with an anti-Rad9 antibody and analyzed for the association with Hus1 and WRN. In the untreated Wt TLK1B expressing cells Rad9 was associated with Hus1 (Fig. 5b) and WRN (Fig. 5c) and this was stimulated upon treatment with HU. However, Mut TLK1B expressing cells showed reduced association of Rad9 with Hus1 (Fig. 5b) and WRN (Fig. 5c), suggesting that the Mut TLK1B reduces the stability of 9-1-1 and its interaction with WRN.

We next wanted to examine if the overexpressed Rad9 S328D mutant (flag tagged) can interact with WRN and Hus1 since this association should occur largely on chromatin. Immunoprecipitation of the overexpressed Rad9 S328D mutant using flag antibody showed that the interaction of mutant Rad9 with WRN and Hus1 was greatly impaired, while the overexpressed wt Rad9 was able to bind normally (Fig. 5e, f). In this case, the addition of HU did not increase the association of wt Rad9 with WRN and Hus1 probably due to the overexpression of the protein.

### Mut TLK1B expressing cells show increased amount of DNA damage

**Fig. 6:**
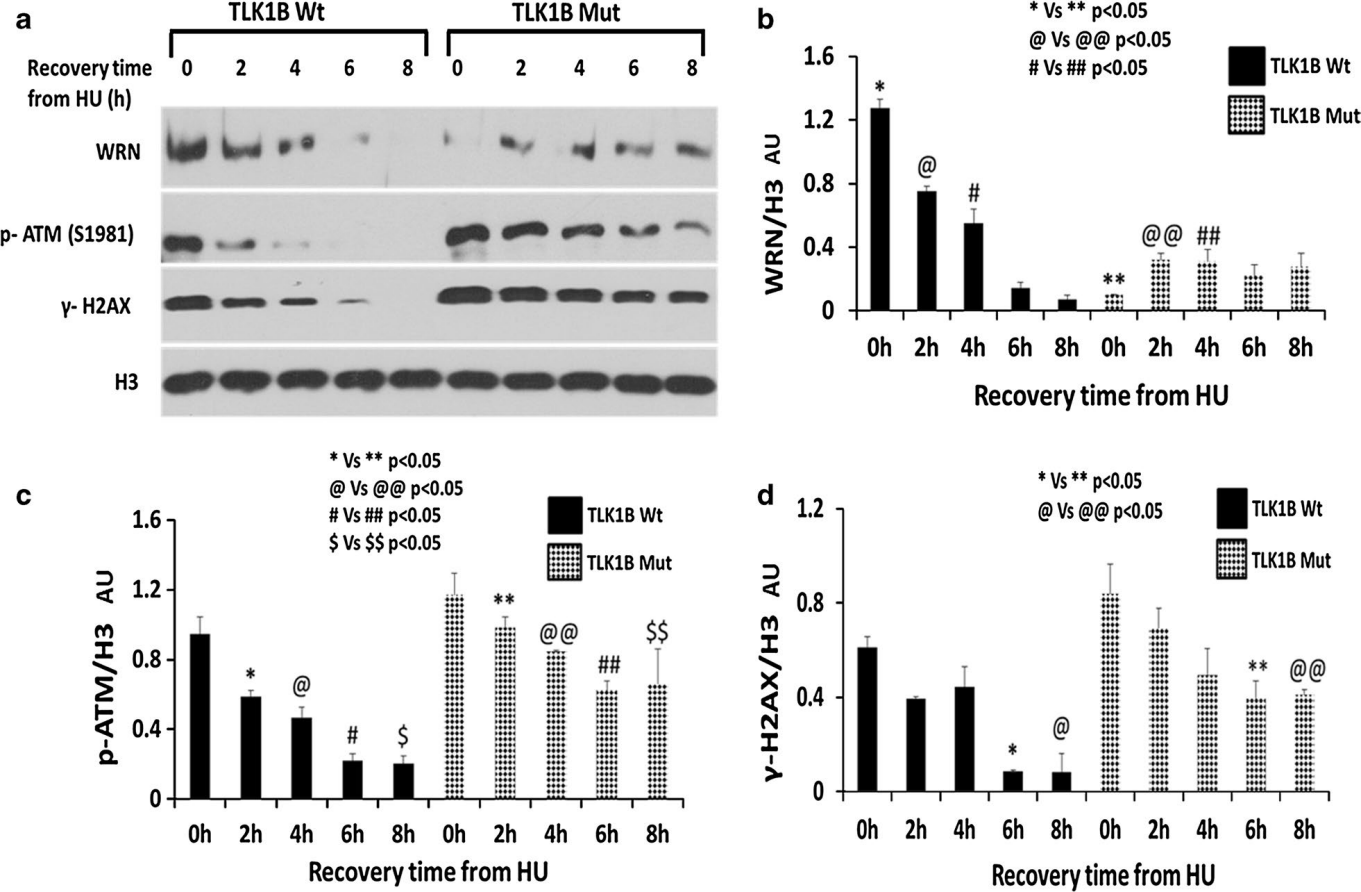
**a** Distribution of WRN, p-ATM (S1981) and γH2AX in the chromatin-bound fraction in Wt and Mut. TLK1B expressing cells in response to treatment and recovery from HU. **b** Less WRN is found in association with chromatin in cells expressing Mut TLK1B particularly at recovery time t = 0. Values are mean ± standard error for n = 3. **c** p-ATM (S1981) associated with chromatin persists during recovery from HU in cells expressing Mut TLK1B. Values are mean ± SE for n = 3. **d** γH2AX persists during recovery from HU in cells expressing Mut TLK1B. Values are mean ± SE for n = 3

It has been shown that the interaction between WRN and the 9-1-1 complex prevent DSB formation at the stalled replication forks [38]. The fraction of WRN bound to chromatin (Fig. 6b) after treatment and release from HU is significantly reduced in the Mut TLK1B expressing cells, particularly at recovery period of 0 h (*p* < 0.05), when maximum number of fork are stalled. We next wanted to examine if the Mut TLK1B expressing cells show an increase in DSBs. In response to DSBs, ATM is autophosphorylated at S1981. This autophosphorylation stabilizes ATM at the DSBs and is a marker of ATM activation. Prolonged treatment with HU generates DSBs. In the chromatin bound fraction of Wt TLK1B expressing cells at the recovery period of 0 h we saw increased levels of p-ATM S1981 (Fig. 6c). However after 4 h of recovery there was a decrease in p-ATM S1981 suggesting that most of the DSBs have been repaired. In comparison, Mut TLK1B expressing cells showed increased levels of p-ATM (Fig. 6c) until 8 h of recovery time (statistically significant). Activated ATM phosphorylates H2AX at S139, which is known as γ-H2AX. In the Wt TLK1B expressing cells γ-H2AX was present only until 4 h of recovery period; however, the Mut TLK1B expressing cells showed increased levels of γ-H2AX till 8 h of recovery time (Fig. 6d). These results suggest that in response to replication stress Mut TLK1B expressing cells have an increase in DSBs that persist beyond 8 h of recovery.

**Fig. 7:**
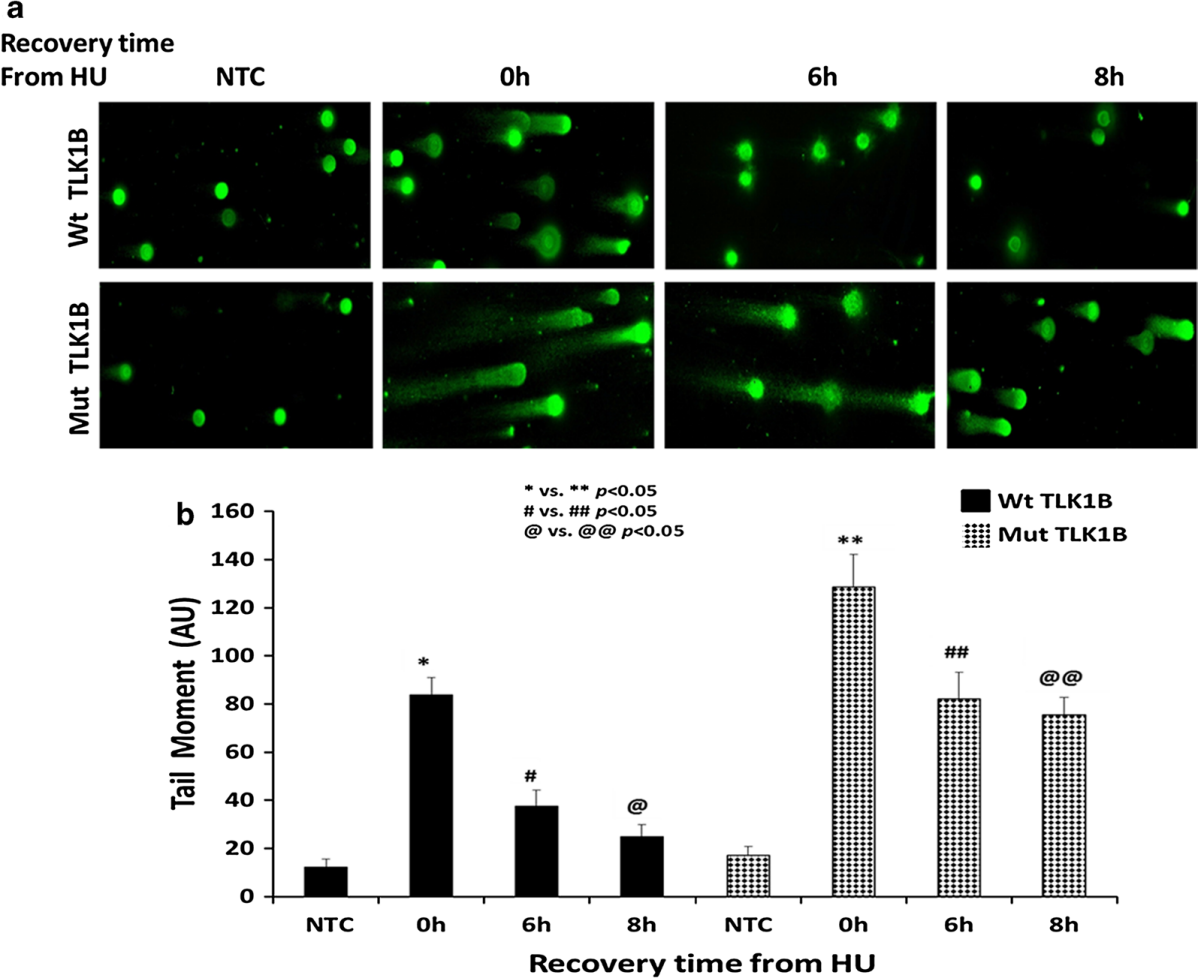
Comet assays was performed to measure the amount of unrepaired DNA damage in untreated cells or cells treated with 2 mM HU and recovery for indicated time points. **a** Representative images of the different time points. **b** Tail moments (tail DNA % × length of tail) as quantified for each cell using ImageJ OpenComet plugin. 50 comet images were measured for each treatment

Activation of ATM is essential for formation of γ-H2AX [39]. Studies from several groups have shown that ATM activation and H2AX phosphorylation can occur even in the absence of DSBs. [40–42]. Since we observed both activation and persistence of ATM and γ-H2AX in Mut TLK1B expressing cells, we probed for presence of DSBs in these cells by comet assays. We observed comets with large tail length and tail moment in the Mut TLK1B expressing cells (Fig. 7) after treatment and recovery from HU, whereas comets were not present at these times in control cells.

### HU treatment of Mut TLK1B expressing cells show a delay in cell cycle progression and recovery

**Fig. 8:**
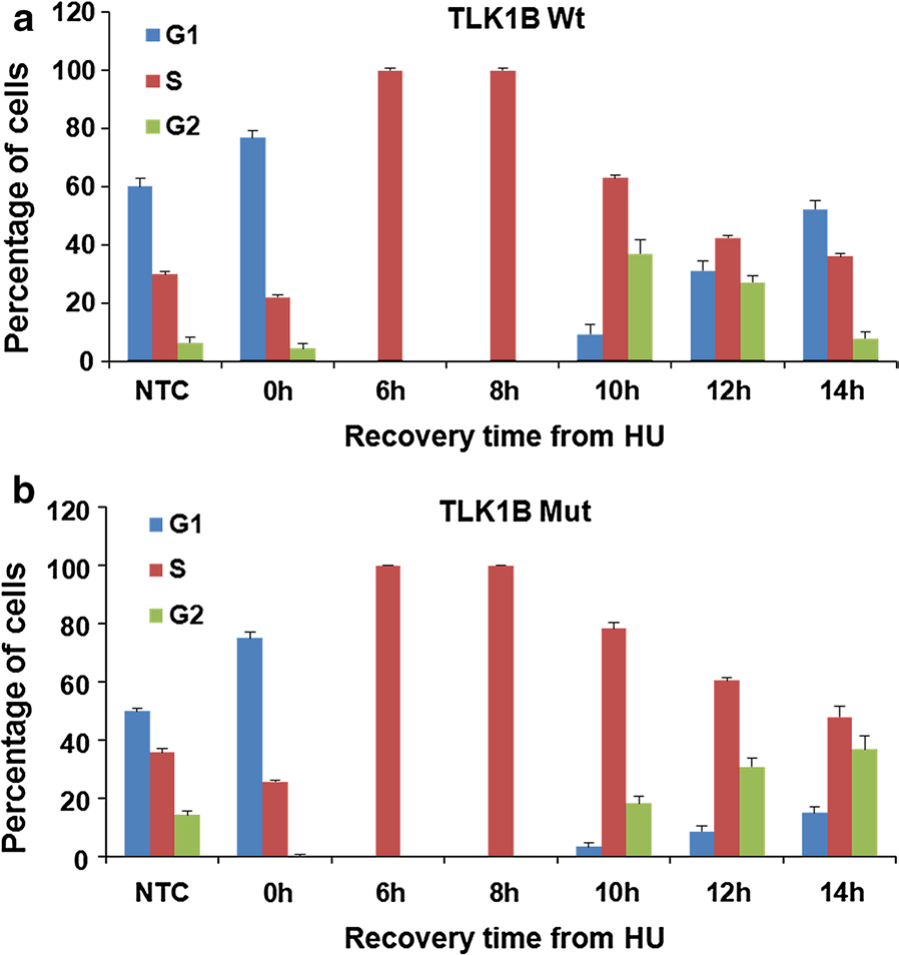
Cell cycle distribution of the cells overexpressing. **a** Wt TLK1B and **b** Mut TLK1B during a time course after release from HU

We wanted to study the effect of this damage resistant kinase on cell cycle progression and recovery from HU induced replication arrest. Treatment with HU causes the cells to accumulate at the G1/S boundary. Washing away the HU allows completion of S-phase and then synchronous entry into G2/M-phase, and then re-entry into the G1-phase of the next cycle. Cells overexpressing Wt TLK1B (Fig. 8a) when released from HU complete S-phase at around 8 h of recovery, synchronously enter into G2/M-phase at around 10 h and then re-enter into the G1 after 12 h. At around 14 h all the Wt cells have recovered. In contrast, Mut TLK1B overexpressing cells (Fig. 8b) remain arrested in the S-phase for 10 h. By 12 h only 7 % of Mut TLK1B expressing cells re-enter back into the G1-phase, in comparison to 35 % of cells expressing Wt TLK1B. By 14 h Mut TLK1B expressing cells still remain in the S- and G2-phase in comparison with the cells expressing Wt TLK1B which mostly re-enter into G-1 phase by that time. Thus, in response to replication stress cells expressing inactivation resistant Mut TLK1B arrest in the S- and G2-phase and show delay in re-entry into the cell cycle. We should stress that in the absence of HU the cell cycle profile is undistinguishable from Wt TLK1B or parental cells (not shown here). Comet assays also showed no presence of DSBs in the absence of HU treatment.

### Mut TLK1B expressing cells display an altered checkpoint control

**Fig. 9:**
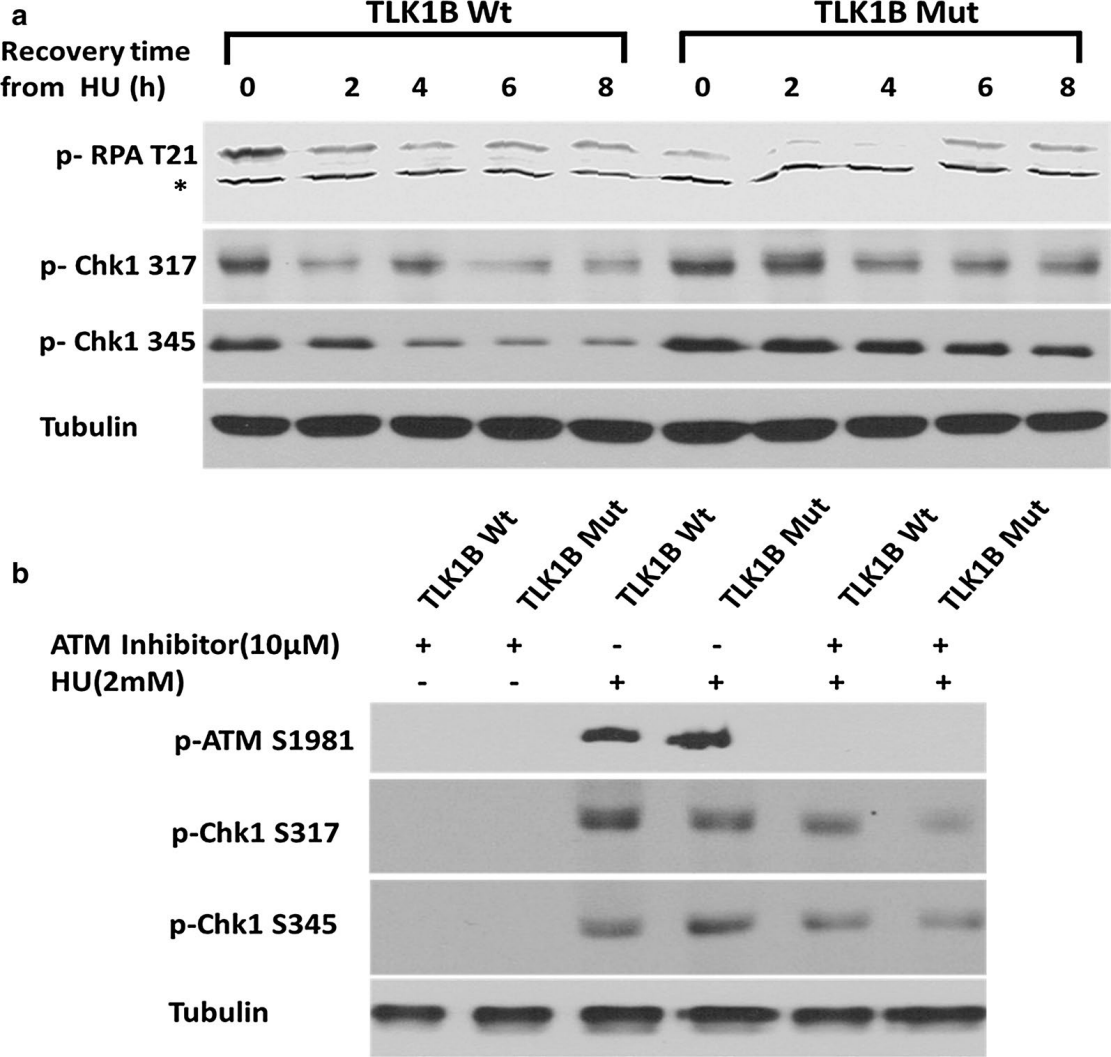
Chk1 activation in Wt and Mut TLK1B expressing cells after treatment and recovery from HU **a** Mut TLK1B expressing cells show reduced phosphorylation of RPA at T21. During recovery from HU, phosphorylation of Chk1 at S317 and S345 persists for 8 h in cells expressing Mut TLK1B, in contrast to Wt expressing cells. **b** Treatment of cells with KU-55933 leads to a reduction of Chk1 phosphorylation in the Mut TLK1B expressing cells

ATR is the primary kinase that phosphorylates RPA2 at T21 after HU treatment [43, 44]. Phosphorylation of RPA by ATR stimulates DNA synthesis and prevents ssDNA accumulation during replication stress [45]. Since less Rad9 was associated with the chromatin in the Mut TLK1B expressing cells, we expected to see a reduced phosphorylation of the ATR substrates in these cells. As expected, we observed a reduced phosphorylation of RPA2 T21 in the Mut TLK1B expressing cells (Fig. 9a). However, we didn’t observe a reduction in Chk1 phosphorylation (Fig. 9a). In Mut TLK1B expressing cells we saw an increased accumulation of DSBs and ATM activation. Since we observed Chk1 phosphorylation in the Mut TLK1B expressing cells, we wanted to examine if this was mediated via ATM. It has been shown that ATM and ATR pathways exhibit a high degree of crosstalk [46–49]. ATM has been shown to phosphorylate Chk1 in vitro and increase in DSB accumulation has been shown to induce ATM mediated phosphorylation of Chk1 [50, 51]. Treatment with a specific ATM inhibitor (KU-55933) showed a slight reduction in Chk1 phosphorylation in the Mut cell line (Fig. 9b), suggesting that the increased Chk1 phosphorylation in these cells could be due to an increase in ATM activity, as shown in Fig. 6. Since we know that ATR is inhibited in these cells due to the lack of chromatin bound Rad9, there is the possibility that part of the Chk1 phosphorylation is instead due to the activity of DNA-PKcs in Mut TLK1B expressing cells [52–54]—this needs to be investigated further.

## Discussion

Tousled Like kinases (TLK) are serine/threonine kinases that play an important role in DNA repair. TLK overexpression is observed in multiple cancers and often corresponds to reduced sensitivity towards radiotherapy or chemotherapy due to the efficient repair in those tumors [32, 55]. Our lab identified TLK1B, which is a splice variant of TLK1 gene from a library of mRNAs that are translationally upregulated by overexpression of translation initiation factor 4E. TLK1B is known to protect cells from genotoxic stress and is translationally upregulated in response to stress and DNA damage via mTOR-eIF4E pathway [13, 56]. At the same time, genotoxic stress leads to Chk1 mediated transient inactivation of TLK [11]. TLK1B promotes repair of damaged DNA in cooperation with Rad9 by facilitating the assembly of repair proteins to the sites of DNA damage. TLKs are the only kinases that phosphorylate Rad9 at S328. Rad9 is aberrantly expressed in prostate, breast, thyroid, skin, lung, and gastric cancers [57, 58]. It plays a major role in cell cycle checkpoint and DNA damage repair. It is essential for genomic stability as frequent chromosomal breakage is observed in cells in which both the Rad9 alleles are inactivated [59, 60]. Rad9, a member of PCNA-like 9-1-1 complex, contains 110 amino acid long C-terminal region that does not share homology with PCNA. This C-terminal region of Rad9 is extensively modified by phosphorylation. Some residues are constitutively phosphorylated while some are transiently phosphorylated in response to DNA damage and cell cycle position [36, 61]. In response to DNA damage, once TLK1B regains its kinase activity it transiently phosphorylates Rad9 at S328. In this study we wanted to elucidate the significance of this phosphorylation.

**Fig. 10:**
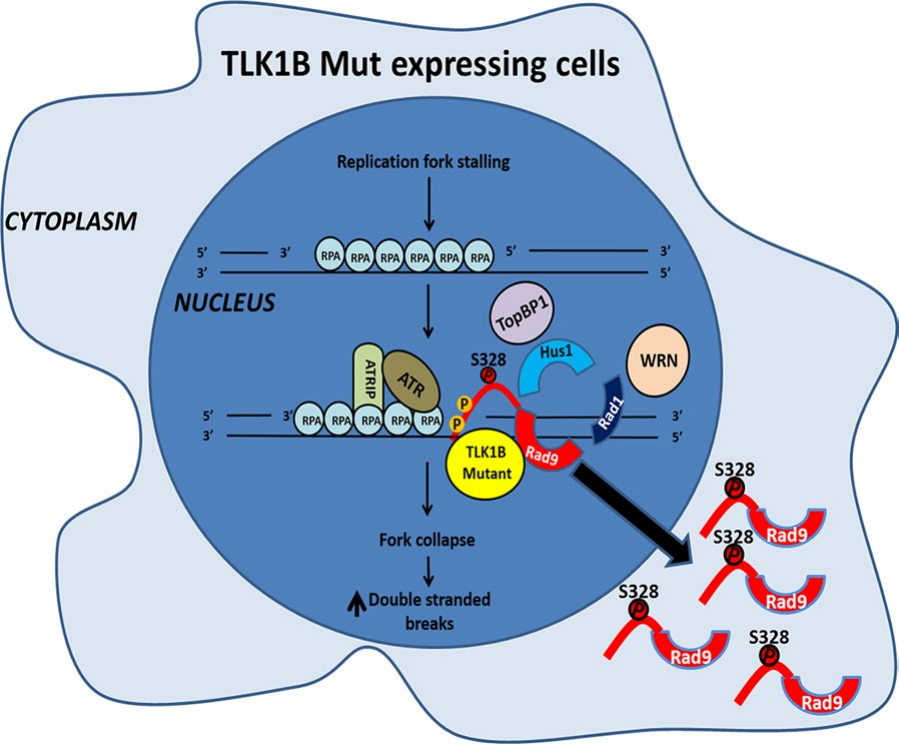
Model for the TLK1B mediated phosphorylation of Rad9 leading to dissociation of 9-1-1 from damage sites and redistribution of Rad9 to the cytoplasm

It has been shown that the stable 9-1-1 complex is required for WRN localization at the stalled replication forks [23], and is necessary for the ATR-dependent phosphorylation of WRN following replication fork stalling [62]. The possible role of the TLK1B mediated phosphorylation of Rad9 on its interaction with WRN was not known. In this work we investigated the significance of the inhibition of TLK1B activity following replication stress and its consequence for Rad9 phosphorylation in relation to the DNA damage response (DDR) and checkpoint recovery. In previous studies using a kinase dead TLK1B we have found that the release of Rad9 from a DSB induced with HO nuclease was delayed well beyond the time required to repair the break [14]. Conversely, in this work we report that constitutive phosphorylation of Rad9 by the mutant TLK1B after release from HU results in its dissociation from chromatin and translocation to the cytoplasm (Fig. 10). Consistent with this model, the Rad9 (S328D) mutant was found exclusively in the cytoplasm and didn’t associate with the chromatin. We also studied the consequences of this effect of inactivation resistant TLK1B mutant on DDR and cell cycle checkpoint. We found that the cells delayed in cell cycle recovery following release from HU and remained in the S- and G2 phase for several more hours compared to the cell expressing WT TLK1B. Our explanation for these results is that premature phosphorylation of Rad9 mediated by the inactivation resistant TLK1B mutant leads to the dissociation of the 9-1-1 complex from sites of damage and stalled forks (Fig. 10). This dissociation leads to fork collapse and generation of DSBs marked by increase in γH2AX and activated ATM. ATM activates Chk1, marked by its phosphorylation at S317 and S345, which mediates cell cycle arrest. The premature dissociation of the 9-1-1 complex from stalled forks in Mut TLK1B expressing cells results in lesser amount of Rad9 associated with Hus1 and WRN. In fact, there was less WRN in association with chromatin after release from HU in Mut TLK1B expressing cells, which would be expected to result in increased fork collapse, as WRN promotes replication fork recovery [62]. Thus, in this study we have identified the significance of Rad9 S328 phosphorylation and have shown that the presence of the damage resistant active TLK1B targets Rad9 to the cytoplasm. We should emphasize that loss of Rad9 from the nucleus can increase the instances of chromosomal breakage leading to genomic instability. Scott Davey’s group reported that TLK1 phosphorylates primarily Rad9(T335) and that plays a key role in cell cycle progression and G2/M checkpoint exit [30]. In our Mut TLK1B overexpressing cells, the phosphorylation of Rad9 (T355) was very weak, but more importantly, we could not see a difference in cytoplasmic/nuclear redistribution when a T355D mutant was used. The accumulation of Rad9 in the cytoplasm is consistent only with the S328 phosphorylation data and the S328D substitution, whereas the T335D phosphomimetic mutation was not sufficient to accumulate Rad9 in the cytoplasm.

## Conclusions

Our collective work has demonstrated that TLK1B acts as a chaperone for Rad9. In the absence of TLK1 kinase activity, i.e., in presence of DNA damage or when a kinase-dead protein is used, we showed that TLK1B promoted the association of Rad9 with a DSB and presumably also SSB. Once the damage is repaired, the kinase activity recovers and then TLK1B phosphorylates Rad9 at S328, promoting its dissociation from 9-1-1 and export to the cytoplasm, thereby mediating the deactivation of the DDR. In conclusion transient inhibition of the kinase after DNA damage is crucial in retaining 9-1-1 at damage sites until repair is complete, and mutations in the TLK1 gene which can activate the kinase may potentially cause accumulation of DSBs.

## Methods

Hydroxyurea (Catalog No. H8627), doxorubicin hydrochloride (Catalog No. D1515), thioridazine hydrochloride (Catalog No. T9025), KU-55933 (Catalog No SML1109) and G418 disulfate salt (Catalog No. A1720) were purchased from Sigma. Dulbecco’s modified Eagle’s medium (DMEM) was obtained from Life Technologies (Catalog No. 12100-046), fetal bovine serum (FBS) was obtained from Atlanta Biologicals (Catalog No. 900108). Enhanced chemiluminescence solution was obtained from Thermo-Scientific (Catalog number No. 32106).

Antibodies used in this study were: rabbit α-Actin (Ab1801, Abcam), rabbit α-Rad9 phospho-328 (AP3225a, Abgent), rabbit α-Chk1 phospho-317 (AP3070a, Abgent), rabbit α-Chk1 phospho-345 (sc-17922, Santa Cruz Biotechnology), goat α-Rad9 (sc-10465, Santa Cruz Biotechnology), mouse α-Rad9 (sc-8324, Santa Cruz Biotechnology), donkey α-goat IgG-HRP (sc-2020, Santa Cruz Biotechnology), rabbit α-TLK1 phospho-695 (4121S, Cell Signaling), α-rabbit IgG-HRP (7074S, Cell Signaling), α-mouse IgG-HRP (7076, Cell Signaling), rabbit α-TLK1 (GTX102891, GeneTex), mouse α- H2A.X phospho-139 (05-636, Millipore), mouse α-Flag (F1804, Sigma) and Rabbit α-Flag (F7425, Sigma).

### Cell culture

HEK293 cells, obtained from ATCC repository, were maintained in DMEM supplemented with 10 % FBS and 1 % penicillin–streptomycin at 37 °C in a humidified incubator with 5 % CO_2_. For ATM inhibitor experiment, cells were pretreated with 10 µM KU-55933 for 2 h. 2 mM HU was then added into the media for 16 h. For inhibition of TLK cells were pretreated with 10 µM Thioridazine hydrochloride (THD) for 2 h before addition of 2 mM HU.

### Cell cycle analysis

HEK293 cells were seeded in T-25 flasks at a density of 2 × 10^5^ cells/flask. Cells were treated with 2 mM HU for 16 h. They were briefly washed with 1× PBS and fresh media was added to the cells. Cells were allowed to recover for indicated time points. Cells were washed with 1× PBS and trypsinized. Cell suspensions were centrifuged at 1000 rpm for 5 min, and pellets were fixed with ethanol and stained with 50 μg/ml propidium iodide (Sigma, Catalog No. P4170). Percentages of cells within each of the cell cycle compartments (G0/G1, S, or G2/M) were determined using a FACS Calibur flow cytometer (Becton–Dickinson).

### Chromatin-bound fractionation

To isolate chromatin, ~10^7^cells cells were resuspended in buffer A (10 mM HEPES, [pH 7.9], 10 mM KCl, 1.5 mM MgCl_2_, 0.34 M sucrose, 10 % glycerol and 1 mM DTT) supplemented with halt EDTA free protease and phosphatase inhibitors (Life Technologies, Catalog No. 78441). Triton X-100 (0.1 %) was added, and the cells were incubated for 5 min on ice. Cytosolic proteins were separated from nuclei by centrifugation (4 min, 1300×*g*). Nuclei were washed once in solution A, and then lysed in solution B (3 mM EDTA, 0.2 mM EGTA and 1 mM dithiothreitol) supplemented with halt EDTA free protease and phosphatase inhibitors for 30 min. Insoluble chromatin was then separated from soluble nuclear proteins by centrifugation (4 min, 1700×*g*), washed once in solution B, and collected by centrifugation (1 min, 10,000×*g*). The final chromatin pellet was resuspended in SDS sample buffer. Samples were sonicated for 15 s. Aliquots of each fraction were separated on sodium dodecyl sulfate–polyacrylamide (SDS-PAGE) gels and blotted onto polyvinylidene difluoride (PVDF) membranes.

### Western blot analysis

Cells were lysed in 1X SDS sample buffer. Lysates were sonicated for 15 s and heated at 100 °C for 5 min. Proteins were separated on 6–12 % SDS-PAGE gels and transferred to PVDF membranes (Millipore). Membranes were incubated with PBS containing 0.05 % Tween 20 and 5 % non-fat dry milk to block non-specific binding and were incubated with primary antibodies; membranes were then incubated with appropriate secondary antibodies conjugated to horseradish peroxidase. Immunoreactive bands were visualized using chemiluminescence reagent.

### Construction of mammalian expression vectors and generation of stable cell lines

We had cDNA of the human TLK1B cloned into the BK-shuttle vector [9]. To sub-clone TLK1B into pIRES2 vector the TLK1B ORF was amplified by PCR with the primers: 5′-GTACCGGAATTCAAAATTATTCAGACTGATCTC-3′; 5′-TAATTAGGATCCTGGAGGAAAGTCAGTAAGTAATTA-3′, containing an *Eco*RI and *a Bam*HI tail respectively. The TLK1B PCR product was sub-cloned in the plasmid pIRES2-EGFP, which was cut with the same enzymes. TLK1B S457A mutant was generated from pIRES2-EGFP using the QuikChange Site-Directed Mutagenesis Kit (Stratagene, Catalog No. 200518) with the following primer: 5′-GAGAAGATCAAAT**G**CTTCAGGAAACCTACAC-3′.

The PCR product was transformed in bacteria, and the presence of the nucleotide substitution was confirmed by DNA sequencing. To generate stable cell lines, HEK293 cells were transfected using Lipofectamine 3000 (Life Technologies, L3000001) as per the manufacturer’s protocol, and stably transfected cells were selected with G418 (500 μg/ml). The pIRES2-EGFP permits the translation of both the TLK1B gene cloned into the multiple cloning site and EGFP from the single bicistronic mRNA. After 30 days of selection in G418, cells expressing high levels of GFP were sorted by flow-cytometry.

We had cDNA of the human Flag-tagged Rad9 cloned into an episomal pREP10 vector. Stable expression of this vector requires expression of full length EBNA-1. To generate stable cell lines we transfected HEK293 c-18 cells (ATCC CRL-10852) which stably express full length EBNA-1. Under G418 selection HEK293 c-18 cells stably express EBNA-1 which is required to maintain pREP10 vector episomally. Maintenance of pREP10 vector requires hygromycin selection.

Rad9 S328D and T355D mutant was generated using the QuikChange Site-Directed Mutagenesis Kit (Stratagene, Catalog No. 200518) with the following primers: 5′-CTGCCCTCCATTTCCCTT**GAC**CCTGGCCCCCAG-3′ (Rad9 S328D)5′- CAGTACAGTGCCTGGG**GAT**CCCCCACCCAAGAAGTTC-3′ (Rad9 T355D).

The PCR product was transformed in bacteria, and the presence of the nucleotide substitutions were confirmed by DNA sequencing. To generate stable cell lines, HEK293 c-18 cells were transfected using Lipofectamine 3000 as per the manufacturer’s protocol, and cells were selected under G418 (250 μg/ml) and hygromycin (50 μg/ml) challenge for 30 days.

### Immunofluorescence

Cells were grown on culture slides. To remove soluble proteins and fix chromatin-bound proteins, cells were pre-extracted in buffer1 (1 % Triton X-100, 10 mM HEPES pH 7.4, 10 mM NaCl and 3 mM MgCl_2_) supplemented with halt EDTA free protease and phosphatase inhibitors for 5 min at 4 °C. Cells were then fixed in 4 % paraformaldehyde for 10 min at 4 °C and then treated in buffer 2 (0.5 % Triton X-100, 20 mM HEPES pH 7.4, 50 mM NaCl, 3 mM MgCl_2_ and 300 mM sucrose) supplemented with halt EDTA free protease and phosphatase inhibitors for 5 min at 4 °C. Cells were then blocked for 1 h in SuperBlock solution.

To look at Rad9 cellular localization cells were fixed with methanol and then rehydrated with PBS and blocked for 1 h in SuperBlock solution. Staining with primary antibody was performed overnight at 4 °C in blocking solution, whereas species specific fluorescein/Texas red conjugated secondary antibody (Vector Labs) was applied for 1 h at RT, followed by counterstaining with DAPI. All the primary antibodies were used at a 1:250 dilution, whereas the secondary antibodies were employed at a 1:500 dilution. Fluorescence images were captured using a Zeiss Axioskop 2 microscope.

### Immunoprecipitation

For co-immunoprecipitation (CoIP) experiment, cells were lysed in the lysis buffer (1 % Triton X-100, 0.5 % Na-Doxycholate, 150 mM NaCl, 2.5 mM MgCl2, 1 mM EGTA, 1 mM EDTA, 20 mM Tris/HCl pH 8.0) supplemented with halt EDTA free protease and phosphatase inhibitors. Cell lysates were sonicated for 20 cycles using Diagenode Bioruptor and then incubated with 250 U of benzonase. In total 1.5 mg of cell lysate was precleared with protein A/G Sepharose beads and then incubated overnight at 4 °C with rabbit polyclonal anti-Rad9 (4 μg; Santa Cruz Biotech) or anti-Rabbit IgG, and tumbled with 70 μl of protein A/G Sepharose beads for 4 h at 4 °C. After extensive washing in CoIP buffer, proteins were eluted by boiling treatment in 2X electrophoresis sample buffer prior to Western blotting analysis.

### Comet assay

Neutral comet assay was performed using comet assay kit from TREVIGEN (Catalog# 4250-050-K) following manufacturer’s instructions. DNA was stained with SYBR Gold and fluorescence images were captured using a Zeiss Axioskop 2 microscope. Tail moments were quantified for each cell using ImageJ OpenComet plugin. 50 comet images were measured for each treatment.

### Statistics

Statistical analysis of data was done using one way analysis of variance (ANOVA) with Sigma Stat statistical software. A *p* value of 0.05 or less was considered significant.

## Additional files

10.1186/s12867-016-0056-x Immunofluorescence was performed as described in methods using the α-Rad9 Ab to detect the cellular distribution of Rad9 in the overexpressed Wt and mut TLK1B expressing cells.
10.1186/s12867-016-0056-xA) Distribution of p-Rad9 (T355) in the cytoplasmic fraction in Wt and Mut TLK1B expressing cells. Note that p-Rad9 (T355) shows an increased cytoplasmic accumulation in Mut TLK1B expressing cells after treatment with HU. It should be noted that Immobilon Western HRP Substrate (Millipore catalog# WBKLS0500) was used which provides high sensitivity. B) Chromatin fractionation assay was performed to examine the cellular distribution of the overexpressed flag tagged Rad9 (T355D) mutant after treatment with 2 mM HU for 16 h and allowing them to recover at indicated timepoints. α-Flag Ab detects specifically the cellular distribution of overexpressed flag tagged Rad9 (T355D) mutant.
