# Supplementary figures and images for: TLK1B mediated phosphorylation of Rad9 regulates its nuclear/cytoplasmic localization and cell cycle checkpoint
Source: BMC Mol Biol. 2016 Feb 9;17:3. doi: 10.1186/s12867-016-0056-x (PMC4746922; doi:10.1186/s12867-016-0056-x)

**SUPPLEMENTAL FIGURE 1:**


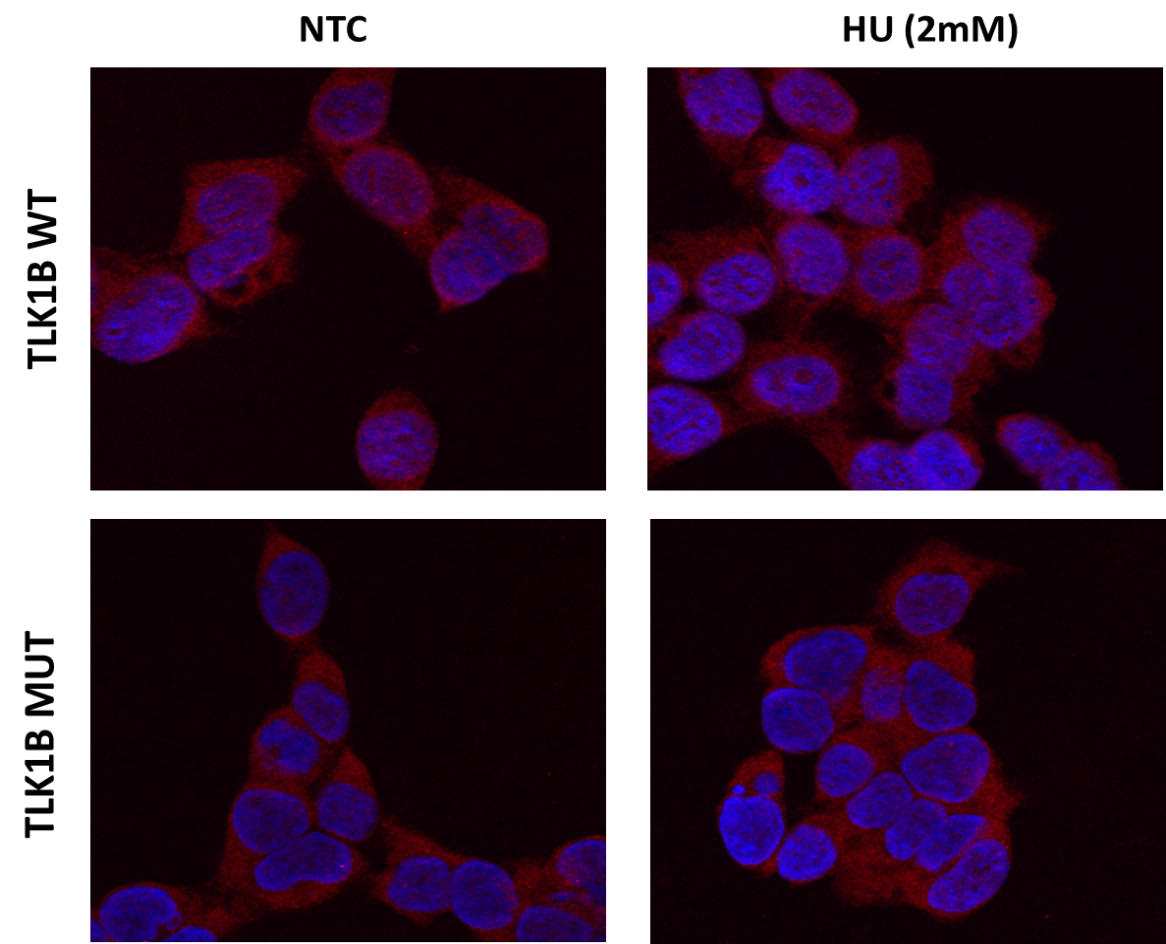

Supplement: Supplementary file 1 — 10.1186/s12867-016-0056-x Immunofluorescence was performed as described in methods using the α-Rad9 Ab to detect the cellular distribution of Rad9 in the overexpressed Wt and mut TLK1B expressing cells. [file 12867_2016_56_MOESM1_ESM.docx]

**SUPPLEMENTAL FIGURE 2:**


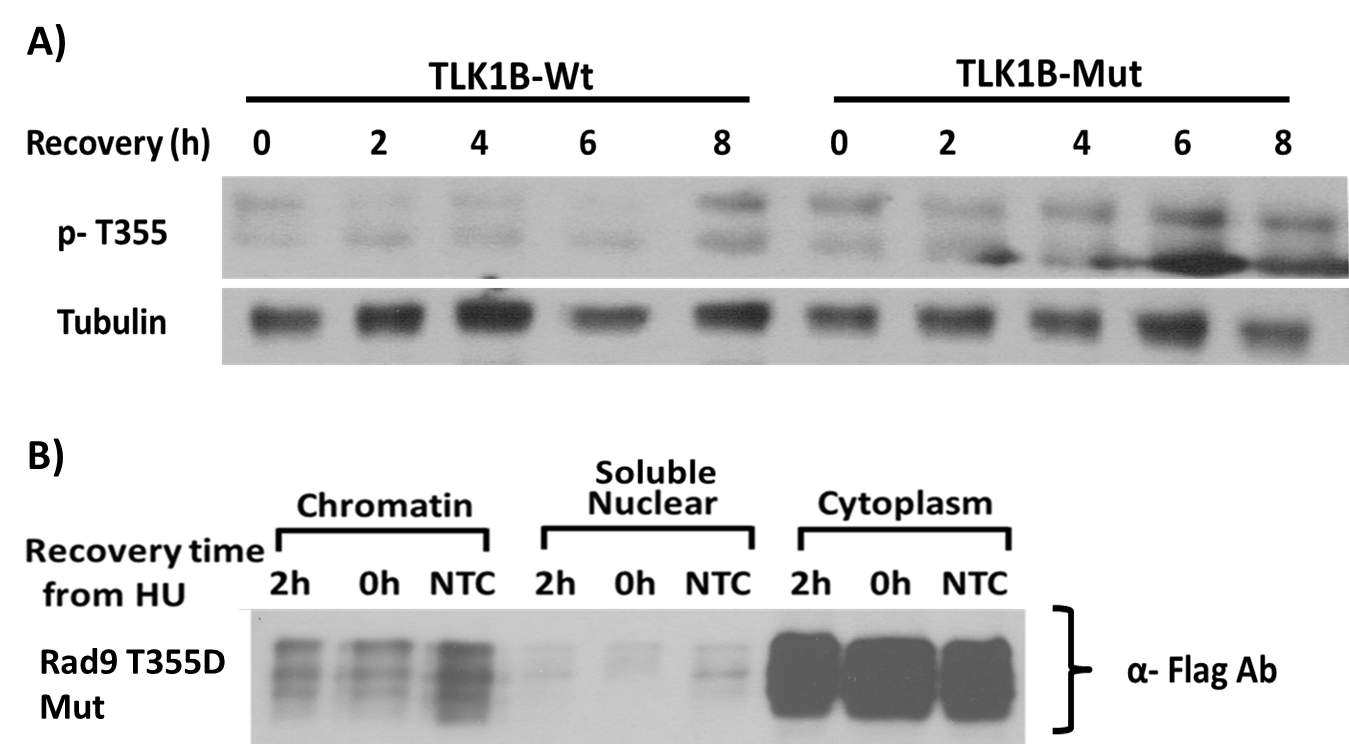

Supplement: Supplementary file 2 — 10.1186/s12867-016-0056-xA) Distribution of p-Rad9 (T355) in the cytoplasmic fraction in Wt and Mut TLK1B expressing cells. Note that p-Rad9 (T355) shows an increased cytoplasmic accumulation in Mut TLK1B expressing cells after treatment with HU. It should be noted that Immobilon Western HRP Substrate (Millipore catalog# WBKLS0500) was used which provides high sensitivity. B) Chromatin fractionation assay was performed to examine the cellular distribution of the overexpressed flag tagged Rad9 (T355D) mutant after treatment with 2 mM HU for 16 h and allowing them to recover at indicated timepoints. α-Flag Ab detects specifically the cellular distribution of overexpressed flag tagged Rad9 (T355D) mutant. [file 12867_2016_56_MOESM2_ESM.docx]
